# Natural History of Tuberculosis: Duration and Fatality of Untreated Pulmonary Tuberculosis in HIV Negative Patients: A Systematic Review

**DOI:** 10.1371/journal.pone.0017601

**Published:** 2011-04-04

**Authors:** Edine W. Tiemersma, Marieke J. van der Werf, Martien W. Borgdorff, Brian G. Williams, Nico J. D. Nagelkerke

**Affiliations:** 1 KNCV Tuberculosis Foundation, The Hague, The Netherlands; 2 Center for Infection and Immunity Amsterdam (CINIMA), Academic Medical Center, University of Amsterdam, Amsterdam, The Netherlands; 3 Cluster Infectious Disease Control, Municipal Health Service (GGD) Amsterdam, Amsterdam, The Netherlands; 4 South African Centre for Epidemiological Modelling and Analysis, Stellenbosch, South Africa; 5 Department of Community Medicine, Faculty of Medicine and Health Sciences, UAE University, Al Ain, United Arab Emirates; McGill University, Canada

## Abstract

**Background:**

The prognosis, specifically the case fatality and duration, of untreated tuberculosis is important as many patients are not correctly diagnosed and therefore receive inadequate or no treatment. Furthermore, duration and case fatality of tuberculosis are key parameters in interpreting epidemiological data.

**Methodology and Principal Findings:**

To estimate the duration and case fatality of untreated pulmonary tuberculosis in HIV negative patients we reviewed studies from the pre-chemotherapy era. Untreated smear-positive tuberculosis among HIV negative individuals has a 10-year case fatality variously reported between 53% and 86%, with a weighted mean of 70%. Ten-year case fatality of culture-positive smear-negative tuberculosis was nowhere reported directly but can be indirectly estimated to be approximately 20%. The duration of tuberculosis from onset to cure or death is approximately 3 years and appears to be similar for smear-positive and smear-negative tuberculosis.

**Conclusions:**

Current models of untreated tuberculosis that assume a total duration of 2 years until self-cure or death underestimate the duration of disease by about one year, but their case fatality estimates of 70% for smear-positive and 20% for culture-positive smear-negative tuberculosis appear to be satisfactory.

## Introduction

Before the advent of chemotherapy, tuberculosis was one of the major causes of death in both Western [1] and also several non-Western countries [2]. While effective chemotherapy for tuberculosis has been available since the 1950s (isoniazid (INH) was introduced in 1952, the less effective para-aminosalicylic acid (PAS) and streptomycin slightly earlier [3]) the prognosis of untreated tuberculosis is still of great importance, as many patients will not receive appropriate treatment because their condition was never properly diagnosed as tuberculosis. For example, both the Cambodian [4] and Vietnamese [5] prevalence survey show that only about 20% of tuberculosis-patients identified in these surveys were on treatment at the time of the survey. This is especially true for smear-negative culture-positive pulmonary cases because in many places in the world Ziehl-Neelsen (Z-N) direct sputum smear, with low sensitivity for paucibacillary disease, is the only available diagnostic tool [6]. Also, many national tuberculosis programmes based on the DOTS (directly observed therapy, short course) strategy only offer free treatment to smear-positive cases in view of their disproportionate role in tuberculosis transmission and thus their large public health impact [7]. In addition, despite the availability of standard chemotherapy, with the recent increases in multi-drug resistant (MDR) and extensively drug resistant (XDR) tuberculosis [8] many patients will have a prognosis that is in all likelihood not very different from untreated tuberculosis. This also holds true for tuberculosis, both drug susceptible and resistant, in HIV-positive patients, most of whom live in Sub-Sahara Africa, where adequate diagnosis and treatment is unavailable in many (especially rural) areas. Since a substantial number of tuberculosis cases will not receive adequate treatment the prognosis in terms of duration and outcome of (untreated) tuberculosis is an important parameter in models used for estimating the disease and mortality burden caused by tuberculosis [9], [10].

The prognosis of untreated tuberculosis is difficult to study these days as leaving patients untreated, especially in a study setting, is unethical. As an alternative, one could consider, as an approximation, the prognosis of multi-drug resistant (MDR) tuberculosis treated with first line drugs. However, MDR-tuberculosis patients may benefit to some extent from first line therapy [11]–[14] and many of these patients may have a history of tuberculosis treatment and may thus suffer from a relapse with secondary (acquired) drug resistance with a prognosis that may differ from that of those who never received tuberculosis treatment. Furthermore, *Mycobacterium tuberculosis* strains resistant to rifampicin and INH might be less fit than drug susceptible strains and therefore lead to longer duration of disease with less mortality [15]. Therefore, to estimate the prognosis of HIV-negative tuberculosis, one inevitably has to rely on data collected in the pre-chemotherapy era even though many of those studies do not meet modern standards.

To estimate the duration and case fatality of untreated pulmonary tuberculosis, we reviewed studies from the pre-chemotherapy era. For tuberculosis in HIV infected patients there are, of course, no data from the pre-chemotherapy era. Thus the only data that are potentially relevant are those on MDR-tuberculosis HIV positive patients treated with (inadequate) first line tuberculosis drugs, as their prognosis would probably be similar to that of untreated HIV positive tuberculosis. However, it is clearly important to distinguish patients by stage of HIV disease and by treatment (ART, type of ART, or not [16]). The complexity of this far exceeds that of estimating the prognosis in HIV- negative patients and requires separate reviews.

We studied the duration until death or self-cure of untreated tuberculosis and 5- and 10-year survival probabilities in representative adult populations (>15 yrs of age) with pulmonary tuberculosis, identifiable as either smear-positive or smear-negative.

## Methods

### Eligibility criteria

Not a single study has measured the duration of disease directly, as this would require an exhaustive ascertainment of incident cases as well as a follow-up to either death, which is easy to establish, or cure, which is more difficult to establish, while withholding treatment, at least for some time. One thus has to rely on indirect information to estimate duration of disease, on the assumption that duration of disease (D) and case fatality (CF) are related to incidence (I), prevalence (P) and mortality (M): D = P/I and CF = M/I [17].

We defined four types of data sources which may contribute information on the natural duration and/or outcome of disease:

1. Follow-up (cohort) studies. Diagnosed patients are individually followed–up over time and their mortality and morbidity experience recorded. Inevitably there is some kind of selection (bias) involved in such studies as they exclude undiagnosed patients. Patients included may be those identified through the health system, or those who attended a specific institution (e.g. sanatorium), or patients may have been identified through a tuberculosis survey. These cohort studies provide key information on CF, but do not generally provide estimates of duration of disease, as the start of the tuberculosis episode is normally unknown and cure is usually not recorded.
2. Prevalence and incidence studies. A comparison between prevalent and incident cases would yield the duration immediately if the population is stable, i.e. no migration. However, if incidence is measured through repeated waves of surveys (instead of recorded continuously), one has to take into account the fact that incident cases occurring in-between surveys, but who recovered or died before the next survey wave, will be missed by the study. Although such studies are ideal for estimating the duration of disease they are less suitable for estimating the CF. In order to obtain an estimate of the CF one needs either follow-up of incident cases or estimates of the frequency with which disease ends in death among those patients for whom the end of disease is observed.
3. Notification and mortality studies. Studies that relate notification to mortality are also relevant. While such studies may provide little information on the duration of disease they do provide data on ultimate outcome (cure versus death) as CF = M/I although one cannot be certain that all incident cases are notified nor that all deaths occur among patients ever notified.
4. Prevalence and mortality studies. These compare the prevalence of tuberculosis to its (annual) mortality, but do not establish the fate of individual patients. To estimate the duration of disease, however, requires knowledge of the CF of (prevalent) tuberculosis cases, as well as an assumption of a stationary epidemiological situation. For then the ratio of the mortality rate and the CF estimates the incidence rate, and one can use the fact that the prevalence equals the product of the incidence and the duration (P = I*D) to obtain the duration. Conversely, estimating the CF would require knowledge of the duration of disease in addition to the prevalence and mortality rate, as the incidence would then equal the prevalence divided by the duration, and the ratio of the mortality and incidence rate would yield the CF.

### Search strategy

We searched PubMed including OldMedline with publications from the early decades of the 20^th^ century up to 17 December 2010 and EMBASE, including references from 1900 until 1966. The search strategy is summarized in Table 1. These searches did, for a variety of reasons (see below), not yield any eligible papers. Therefore, additionally a snowball sampling method was applied, using reference lists of various papers and books, starting with Hans Rieder's book “Epidemiological Basis of Tuberculosis Control” [18], supplemented with literature identified from the authors' personal libraries [19]–[23]. We also asked the members of the tuberculosis expert group of the Global Burden of Diseases, Injuries, and Risk factors study (see Acknowledgements for names) for suitable references. For practical reasons, we only included papers in English, French, German, Spanish and Dutch. Papers in other languages with English table and figure legends as well as an English summary were also included.

**Table 1:** Search strategies used for searching electronic databases.

Database	PubMed*	Old Medline**	Embase†
Period included	1-1-1954 – 17-12-2010	Start – 31-12-1953	Start – 1966
Mesh terms included	Tuberculosis, Prognosis, Mortality	Tuberculosis, Prognosis, Mortality	Tuberculosis, Prognosis, Mortality, Survival, Fatality
Free text included (all fields)	Tuberculosis, Prognosis, Mortality, Survival, Fatality, Untreated	Tuberculosis, Prognosis, Mortality, Survival, Fatality	
Free text included (title/abstract only)	Course	Course	Course
Free text included (title only)	Course	Course	Tuberculosis, Prognosis, Mortality, Survival, Fatality
Number of references retrieved	196	591	1093
Number of references minus duplicates‡	196	537	827

*‘tuberculosis’ (either as Mesh heading or as free text) and ‘untreated’ and one of the other terms (as Mesh term or as free text) were searched for.

**‘tuberculosis’ (either as Mesh heading or as free text) and one of the other terms (as Mesh term or as free text) were searched for.

† ‘tuberculosis’ either as subject heading or as free text in title and ‘course’ as free text in title or abstract or one of the other terms as subject heading or as free text in title.

‡ Occuring as duplicate either within search, with searches in other electronic databases, or with snowball sample.

### Study selection

All references were first screened independently by two authors (ET and NN) on title and, if no title was available, in the snowball sampling method, on reference in the text to assess whether they potentially assessed the prognosis of untreated pulmonary tuberculosis in representative adult populations. Of potentially eligible papers, if available, abstracts were subsequently assessed for eligibility using the same strategy. If no abstract was available, papers were accessed in full text. Among the identified sources we selected those that would potentially provide estimates of CF and/or duration of pulmonary tuberculosis in adults (≥15 years) by any of the four methods outlined above. Studies were included provided: a) their methodology was sound (e.g. (near-to-)complete follow-up or making use of actuarial methods), considering populations that can be considered as more or less ‘population-based’ (thus not including only specific population subgroups or pre-selecting certain categories of patients), b) they contained original data (i.e., no editorials, opinion papers, minutes; reviews were only included if the literature these referred to was not found), c) we could decide whether patients included were smear-positive or smear-negative but culture-positive; in studies where patients were described as having “open” tuberculosis or “bacillary tuberculosis” before 1930 (when culture became available) we assumed that these patients were smear-positive, d) description of the available data was sufficient to enable calculation 5- and/or 10-year survival probabilities or disease duration, and e) the study population was not treated with chemotherapy or was treated with probably or proven ineffective therapy (e.g. collapse therapy, lung resection, short duration mono-drug therapy, etc.).

### Data extraction

Eligibility and data extracted from all eligible sources were checked and discussed by two authors (NN and ET) using the criteria described above. The data sources were reviewed and summarized with respect to their information regarding the duration and outcome of untreated tuberculosis, and CF. Discrepancies between authors with respect to extracted data were resolved by discussing the differences and independently re-reviewing the data.

### Methodological considerations

There are some important limitations to studying the duration and CF of untreated tuberculosis, since many of the included studies do not meet modern research standards. For example, the case definition, the onset of disease, or the beginning of follow-up in cohort studies (onset of symptoms, sputum positivity) are often ill-defined or poorly described in older publications, and many cases included in those studies would not meet modern diagnostic standards.

A large number of studies are based on passive case finding, which inevitably entails some selection bias, as diagnosed cases may well differ from undiagnosed ones. Some studies are limited to hospitalized (sanatoria) cases and therefore presumably exclude both the mildest and the most severe cases, as some of the latter probably died before they could have been hospitalized.

An additional methodological problem constitutes the way cases have been classified in old studies. Using the distinction of pulmonary tuberculosis into sputum smear-positive (smear-positive) and sputum smear-negative (smear-negative) cases, the most common classification used today, we must assume (highly unrealistically) that the sensitivity and specificity of direct smear has not changed. Especially the diagnosis of smear-negative cases is problematic as culture using the Löwenstein-Jensen (L-J) medium did not become available until the 1930s [24], [25] and thus all Z-N smear-negative tuberculosis was diagnosed on the basis of radiology and/or symptoms with uncertain specificity [26]. In some publications cases are reported as having “open” tuberculosis without explicit definition. This presumably depends on various non-standardized Z-N like procedures of directly demonstrating *M. tuberculosis* in sputum. A comparison between sputum smear microscopy used in those days with that currently in use is not available. Another methodological problem, also affecting many modern studies on tuberculosis, is the implicit assumption that pulmonary tuberculosis can reliably be classified as either smear-positive or smear-negative and that no transitions between these categories take place. This is almost certainly untrue, if only because of the poor sensitivity of sputum smear and its dependence on factors such as the number of repeat smears [14]. Yet, it is well established that many smear-positive patients who become smear-negative in the absence of adequate treatment subsequently relapse and become smear-positive again [12]. Whether they are still culture-positive while being smear-negative or temporarily “cured” (i.e. culture-negative) is largely unknown. Presumably, some smear-negative patients who die will become smear-positive prior to death, vitiating the assumption of stable categories. Yet how common this is, remains unknown. Nevertheless, the classification into smear-positive and smear-negative has become so widely established, and is so much part of the methodology of estimating the burden of tuberculosis, that it is impossible to avoid it.

A further methodological pitfall is that by combining different estimates one makes the implicit, and untested, assumption that the natural history of tuberculosis does not differ significantly among countries and periods. However, the risk of infection with *M. tuberculosis* and progression to tuberculosis disease is influenced by host factors and especially risk of progression depends on the hosts' immune status, which may be reduced due to concomitant HIV infection, diabetes, and other underlying diseases [27], [28]. Given these methodological challenges, it is clear that only by combining, often in an ad-hoc fashion, different sources of information can one probably get somewhat adequate or reasonable estimates of the “correct” duration and case fatality (CF) of various types of tuberculosis.

### Summary measures and synthesis of results

Data were extracted into Excel sheets and survival probabilities re-calculated and provided with accompanying 95% Greenwood confidence intervals using the original paper's life table's information. Where insufficient details were available to recalculate survival probabilities, estimates as calculated by the studies' authors were taken. Duration of active pulmonary tuberculosis disease from diagnosis till death or cure could be assessed from two studies with a very different study design [20], [29].

Because of the above-described methodological problems with combining the results of such diverse studies, we did not attempt to do a formal meta-analysis here.

## Results

### Study selection

**Figure 1:**
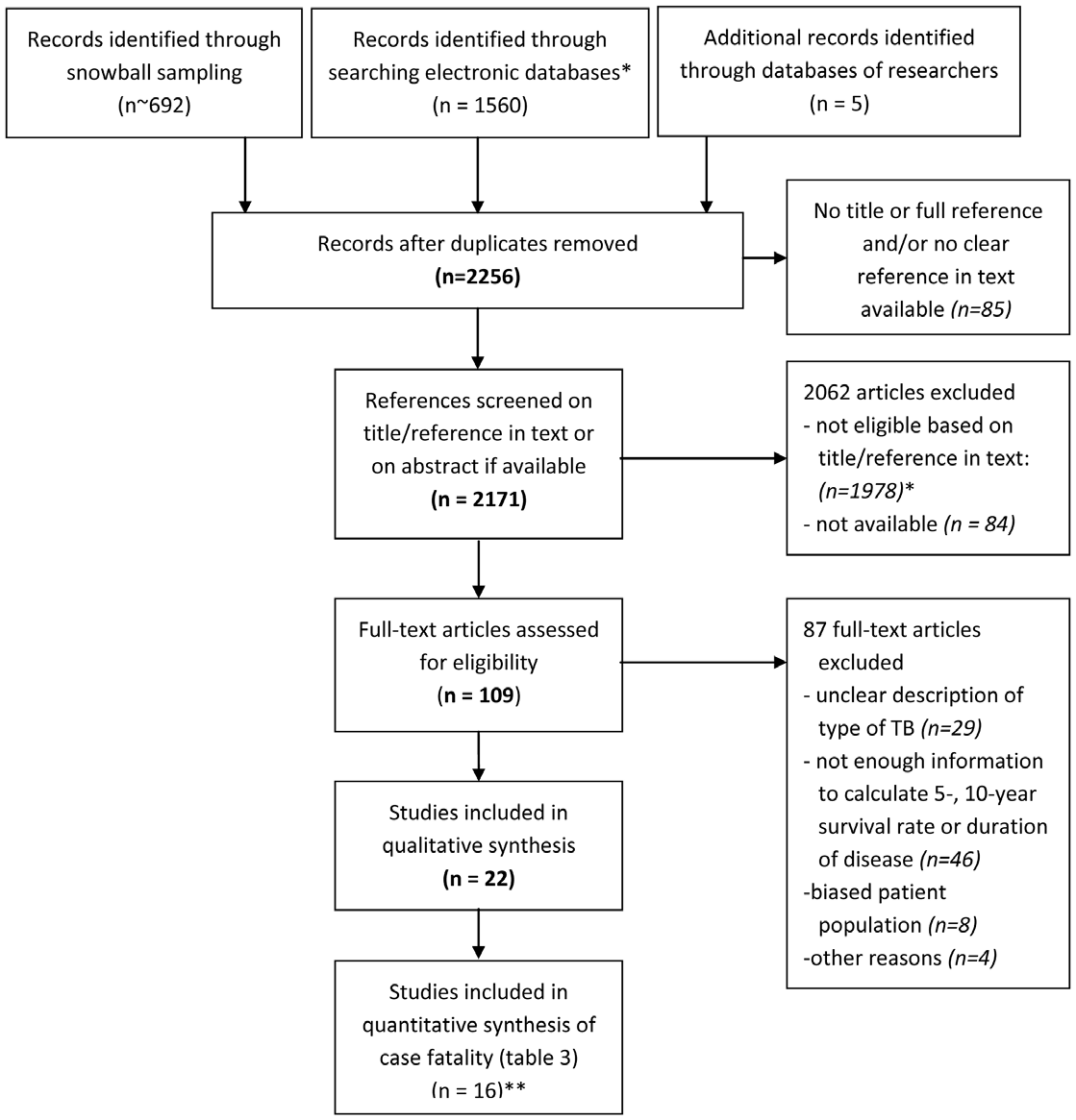
Selection of papers. Flowchart schematically showing inclusion and exclusion of papers. Those marked with a * were excluded either because they were referred to at places in the text that did not discuss duration of tuberculosis, tuberculosis mortality, case fatality, life tables or natural history, or because the title indicated that the paper was not about one of these topics; ** for two of these, data were included to the extent mentioned by Berg [115] (see legend of Table 3).

Using the methods described above we identified a wide range of studies on the prognosis of tuberculosis in the absence of chemotherapy (Figure 1). In total, 2256 references were identified of which 2171 (96%) were screened on title, abstract and/or reference in the text. Of the 193 references selected for full-text reading, 84, i.e. 43% (Note that.) were not available in consulted libraries. However, 32 of these references most probably do not contain any useful information, as they had a very general title including only “tuberculosis” and “mortality” or “research” or “annual report” and appeared in regional journals or were old text books. Another 87 were excluded after reading because they contained no original data [30]–[38] or the selection [39]–[46], description and/or classification of the patients included [21]–[23], [47]–[72], such as the number of smear-positive patients, or (the description of) the available data [73]–[109] were either insufficient, too rudimentary or different from current practice to be useful to us (Figure 1). For example, Elderton and Perry [47], [48] classified patients as “incipient”, “moderately advanced”, “arrested” etc. without providing sufficient details about these patients for us to decide what the operational definition of such classification may have been nor whether these patients were in all likelihood smear-positive or smear-negative culture-positive, or neither. Other authors (*e.g.*, [55], [60]) classified tuberculosis according to three stages defined by Turban. Four papers were excluded because all or part of the patients were treated with chemotherapy. Most of these papers also did not contain sufficient follow-up time nor details to calculate 5-year survival or duration of disease [10], [110]–[112].

### Description of included studies

The sources we considered relevant to the natural (pre-chemotherapy) history of tuberculosis are listed in Table 2. The data sources cover different periods and different countries, but except for two studies [29], [113], all are from the pre-chemotherapy era. All included both sexes. Although sanatorium treatment and surgical therapy were available, these are unlikely to have affected mortality by much [114]. The type of patients included was highly variable in terms of diagnostic criteria (as explained above, diagnostic criteria were often unclear) and age composition (if reported). For example, the age distribution of the population included in the study of Berg was 36%, 50% and 14% for men in the age groups 15–24, 25–44, and 45 years and older, and 43%, 50% and 7% for women [115], whereas that of Drolet's population was 23%, 45%, 33% and 36%, 46% and 18% for men and women respectively [116].

**Table 2:** Overview of studies included in our review*.

Study	Design	Country	Type of Subjects	Period patients identified	N
Hartley *et al.* [118] **,†	Cohort	UK	Cases treated at Brompton Hospital with open tuberculosis	1905–1914	3,326
Sinding-Larsen [119]	Cohort	Denmark	Sanatorium patients with open tuberculosis	1907–1931	1,114
Trail and Stockman [117] **	Cohort	UK	Sanatorium patients with bacillary and abacillary tuberculosis	1911–1928	2,625
Backer [120]	Cohort	Norway	Dispensary material of patients with bacillary and abacillary tuberculosis	1911–1930	2,312
Krebs [121] &	Cohort	Switzerland	Sanatorium patients with open and closed tuberculosis	1912–1927	1,787
Tattersall [123], [124]	Cohort	UK	Dispensary material from smear-positive patients	1914–1940	1,192
Magnusson [125]	Cohort	Iceland	Sanatorium patients with open and closed tuberculosis	1916–1935	792 examined, 379 with open and 413 with closed tuberculosis
Rutledge and Crouch [19]	Cohort	USA	Discharged sanatorium patients with bacillary and abacillary tuberculosis	Not stated, prior to 1919	1,654
Münchbach [126]	Cohort	Germany	Sanatorium patients, with open tuberculosis	1920–1927	3,966
Braeuning and Neisen [127], [128]	Cohort	Poland (then Germany)	Dispensary material of bacillary/open tuberculosis patients	1920–1921, 1927	951
Griep [129]	Retrospective cohort	The Netherlands	Notified cases with open tuberculosis	1920–1938	1,846
Baart de la Faille [130] ‡	Cohort	The Netherlands	Sanatorium patients, with open and closed tuberculosis	1922–1935	3,615 (1,131 smear-positive at least once; 534 smear-positive at discharge)
Buhl and Nyboe [131]	Cohort	Denmark	Notified cases with bacillary tuberculosis	(here) 1925–1929	314
Lindhardt [132]	Cohort	Denmark	Notified cases	1925–1934	5,432 smear-positive cases
Berg [115]	Cohort(s)	Sweden	All diagnosed open tuberculosis patients	1928-1934†	2,042
Thompson [133]	Cohort	UK	All diagnosed smear-positive patients	1928–1938	406
National Tuberculosis Institute (NTI), Bangalore [29]	Successive waves of surveys, prevalence and incidence	India	Active case-finding, smear-positive and/or culture-positive tuberculosis	1961–1968	166,140 examined, 627 with tuberculosis
Pamra *et al.* [113]	Successive waves of surveys, prevalence and incidence	India	Active case-finding, smear-positive and/or culture-positive tuberculosis	1962–1970	21,344–24,808¶ examined, 142 with tuberculosis
Drolet [116]	Notification and mortality	USA and UK	Notified cases with pulmonary tuberculosis (not further specified)	1915–1935	299,244 (parts of USA), 323,870 (UK)
Braeuning [134]	Notification and mortality	Poland (then Germany)	Notified cases with open pulmonary tuberculosis and deaths from tuberculosis	1925–1929	264,500 (annual average)
Framingham Com-munity Health & Tuberculosis Demon-stration [20], [135]–[137]	Community study; prevalence and mortality	USA	Community active and passive case finding of tuberculosis (not specified)	1916–1925	Not precisely given

*Abbreviations used in this table: UK, United Kingdom; USA, United States of America; culture-positive, Löwenstein-Jensen medium culture-positive.

**as reported by Berg [115], since original paper was not available.

† only the years of which least biased data (according to Berg's [115] opinion) were available are included here.

‡ Smear-negative tuberculosis was defined as growth of mycobacteria on Malachite-green culture whereas no bacilli were identified in the patient's sputum.

& Data re-analyzed by Fürth [122], who included the 1464 patients (995 with open and 469 with closed tuberculosis) who were followed for at least 5 years after discharge.

¶ Depending on survey wave (first survey had 21,344 participants, fourth and last had 24,808 participants).

#### Follow-up studies

1. Berg's study [115] is probably the most comprehensive study of all the (retrospective) follow-up studies and has tried to include all patients (including those ascertained after death) with “open” tuberculosis from Gothenburg (Sweden) diagnosed between 1928 and 1934. He followed all patients who were ever found to have bacilli in sputum from diagnosis of tuberculosis. He identified various difficulties and biases (e.g. “ascertainment” biases) in doing so. Berg also reviewed earlier studies on the prognosis of tuberculosis and open tuberculosis more specifically. However, the starting point of follow-up of most of these patients is unclear and the studies usually included highly selected patients (e.g. sanatorium, tuberculosis dispensary), and are thus less representative than Berg's own material from Gothenburg [115]. We included the relevant studies that were not available to us in full text (Trail and Stockman (1931) [117], and Hartley, Wingfield and Burrows (1935) [118]), to the extent summarized by Berg [115]. Trail and Stockman carried out a cohort study in the UK among patients of the King Edward VII sanatorium in Midhurst (UK). Hartley and colleagues did a retrospective cohort study of cases treated for tuberculosis at Brompton Hospital. Only the pre-war (World War I) period is presented here, as Berg considered the results of the period 1915-1931 being less representative.

2. Sinding-Larsen [119] did a cohort study in Denmark among sanatorium patients, with the objective of evaluating the impact of collapse therapy.

3. Backer [120] followed patients notified to the Board of Health in Oslo, Norway, between 1911 and 1920 until 1931 and reported survival from date of notification, not date of diagnosis.

4. Krebs [121] considered pulmonary tuberculosis patients discharged from Barmelweid sanatorium in Switzerland treated from its opening in 1912 up to 1927. In his report patients are categorized according to different categories/stages, including whether tuberculosis is open or closed but he does not clarify the exact definitions of open and closed tuberculosis. It is also unclear whether all closed tuberculosis patients would meet the current definition of smear-negative culture-positive tuberculosis. Probably, the study included patients diagnosed on the basis of chest radiographs or clinical symptoms, as L-J medium was not yet available. Five- and 10-year mortality rates of all 1464 patients who were followed for at least 5 years (discharged between 1912 and 1924) were re-calculated by Fürth [122].

5. Tattersall [123], [124] included sputum-positive cases attending Reading (UK) dispensary between 1914 and 1940 from the time of their diagnosis until death or up to 31 December 1945.

6. Magnusson [125] studied cases admitted for treatment at the Vifillsstadir Sanatorium in Reykjavik, Iceland, recruited between 1916 and 1923 with a subsequent follow-up time reaching up to 1935. Cases of ‘closed’ and open tuberculosis were reported separately.

7. Rutledge and Crouch [19] reported on the follow up of tuberculosis patients discharged from a particular sanatorium in the United States of America (USA). Smear-positive and smear-negative (note: not necessarily culture-positive) cases were reported separately.

8. Münchbach [126] included sanatorium patients with open bacillary tuberculosis, which should probably be interpreted as smear-positive tuberculosis.

9. Braeuning and Neisen [127], [128] included tuberculosis dispensary patients from Szczecin, Poland (then known as Stettin, Germany) from two periods, 1920-21 and 1927-28 from the date of their first positive sputum.

10. Griep [129] followed-up all notified cases of open pulmonary tuberculosis occurring in The Hague, The Netherlands during a 18-year period (1920-1937). Although cultures were being performed, only those who had at least one positive sputum smear were included in his analyses. He estimated that about 62% of all tuberculosis patients were notified, with overrepresentation of those in the lowest socio-economic classes, since those in higher classes probably sought private care.

11. Baart de la Faille [130] explored the outcome of tuberculosis cases hospitalized in the Sanatorium “Berg en Bosch” in The Netherlands. He distinguished three different groups of patients based on sputum smear results at admission and during the last two months before discharge: positive/positive, positive/negative and negative/negative patients. Cultures were being done from 1931 onwards and smear-negative culture-positive patients were added to the negative/negative group, this group thus being a mixture of culture-positive and culture-negative patients. Results from 1936 show that 30% of the negative/negative group in fact had negative smear(s) but one or more positive cultures.

12. Buhl and Nyboe [131] reported on mortality among Danish tuberculosis patients diagnosed between 1925 and 1954. Only patients for whom bacilli had been demonstrated in sputum or gastric washings were included. However, it is not stated by which method bacilli were demonstrated. We therefore only used data from patients diagnosed between 1925 and 1929 (N = 314) as for this period L-J culture was not available yet and all the patients must have been smear-positive. The decline in mortality that they observe during the pre-chemotherapy era suggests that after 1930 some patients were smear-negative culture-positive.

13. Lindhardt [132] reported on tuberculosis mortality in Denmark between 1925 and 1934. All notified cases and all notified smear-positive cases were reported separately. As the category “all cases” may include cases for whom no smear result was available (in addition to smear-negative patients), we only considered smear-positive cases.

14. Thompson [133] included all sputum-positive tuberculosis patients occurring in a compact industrial area in Middlesex County, UK, diagnosed between 1928 and 1938.

#### Prevalence and incidence studies

The study of the National Tuberculosis Institute, Bangalore, India (NTI) [29] involved a series of 4 waves of community surveys in the South of India. The study clearly documents its (more modern) methods and is based on systematic surveys. Pamra [113] and colleagues used very similar methodology in four survey waves following the National Sample Survey in New Delhi. Both studies looked at the (bacteriological) status of survivors during follow-up survey waves and included patients with any chest radiograph abnormalities (screening) who were either positive on direct smear (Z-N/fluorescence microscopy) and/or L-J culture. As such, these are the only studies that included smear-negative, culture-positive patients. In the NTI study, the fractions of smear-negative culture-positive patients were 53.4%, 53.0%, 55.1% and 41.4% in the subsequent 4 surveys. In the other study, the fraction of smear-negative culture-positive cases was lower in the first two surveys (24% and 30%) and similar in the subsequent surveys [113], which may suggest changes in tuberculosis epidemiology, capturing cases earlier in the development of active tuberculosis, or in practice of culturing.

Unfortunately, the reporting of both studies leaves much to be desired. For example, prognosis (death or cure) is not presented broken down by Z-N status (i.e. for smear-positive and smear-negative separately). Moreover, Pamra and colleagues did not give any information about treatment of tuberculosis [113], whereas the NTI study reported that no organized anti-tuberculous treatment was available in the area, and that the study did not provide chemotherapy (except for one month of INH monotherapy at the second and third survey) which was highly unethical given the fact that effective treatment was available at the time of the study. INH was definitely available to some patients in that part of India as the authors discovered a high percentage of INH drug resistance, which again clearly indicates that patients could have been provided with full chemotherapy in this study. In all likelihood treatment was only adequate in some exceptional cases and otherwise of such a low quality that its impact can be ignored [29].

#### Notification and mortality studies

Drolet [116] reported overall mortality ratios (i.e. the ratio of mortality to notification as reported by the departments of Health of the various cities and states in the USA, and the Ministry of Health in the case of the UK) for New York (pulmonary), Chicago (all forms), Detroit (pulmonary), New Jersey (all forms), Philadelphia (all forms, including childhood tuberculosis), Massachusetts (pulmonary), and England and Wales (pulmonary) during the period 1915-35. Braeuning [134] similarly reported population rates, notification rates of new ‘open’ tuberculosis cases and tuberculosis mortality in Stettin between 1925 and 1929.

#### Prevalence and mortality studies

The Framingham Community Health and Demonstration project [20], [135]–[137] was an extensive community based project on tuberculosis epidemiology and prevention initiated in 1916 in the same community that later became the focus of the famous Framingham Heart Study. Several publications report on its findings. Although we did not identify any systematic follow-up of patients, data on the relationship between prevalence and mortality are provided.

### Analysis of Case Fatality

#### Follow-up studies

Direct estimates are available from cohort studies. Table 3 shows 5- and 10-year survival rates from all cohort studies considered in this review. Only one study [125] provided follow-up findings for periods of more than 10 years and showed that mortality rate declined with time since diagnosis. Between 10 and 20 years, mortality for both open and closed tuberculosis dropped to 3.4%, which must have been close to the mortality of non-tuberculous persons. Thus, it seems plausible to assume that almost all mortality will occur within 10 years of onset of disease or diagnosis. Even if the mortality rate and self-cure rate (µ and γ respectively) were constant, i.e. independent of time since onset of disease, the fraction (self) cured among those still alive after 10 years would be (γ/(γ+µ))(1-exp(-(γ+µ)10))/{(γ/(γ+µ))(1-exp(-(γ+µ)10))+exp(-(γ+µ)10)} (which will be close to 1 for values of γ and µ that are consistent with observed 5- and 10-year CF of approximately 59% and 70% respectively (as for smear-positive tuberculosis, see below).

**Table 3:** Survival rates for open (smear-positive) and closed (smear-negative, diagnosed in various ways including chest X-ray) pulmonary tuberculosis.

Study	Number of participants under observation	5-year survival (95% CI)	10-year survival (95% CI)
**Smear-positive/open tuberculosis**			
Hartley [118] *	3326	58% (56%–60%)	-
Sinding-Larsen [119]	1114	57% (54%–60%)	47% (44%–50%)
Trail & Stockman [117] *	2615	50% (48%–52%)	34% (32%–36%)
Backer [120]	2312	35% (33%–37%)	21% (19%–23%)
Fürth [122], re-analyzing data collected by Krebs [121] †	996	30% (27%–33%)	19% (17%–22%)
Tattersall [123]; smear-positive	1082	Not reported	23% (21%–26%)
Magnusson [125]	379	37% (33%–43%)‡	27% (23%–32%)
Rutledge & Crouch [19]	511	39% (35%–43%)	-
Münchbach [126]	3966	50% (48%–52%)	-
Braeuning & Neisen [127]	607	25% (22%–29%)	18% (15%–21%)
Griep; smear-positive [129] §	975	51% (48%–54%)	34% (31%–37%)
Baart de la Faille [130]; smear-positive‡	534	38% (34%–42%)	29% (25%–33%)
Buhl & Nyboe [131]	314	45% (39%–51%)	34% (29%–40%)
Lindhardt; only smear-positive [132]	11,797	43% (42%–44%)	-
Berg [115] ¶	2042	42% (40%–44%)	29% (27%–31%)
Thompson; only smear-positive [133]	406	27% (23%–32%)	14% (11%–18%)
**Smear-negative/closed tuberculosis**			
Fürth [122], re-analyzing data collected by Krebs [121] †	469	88% (85%–91%)	78% (74%–82%)
Magnusson [125]	413	92% (89%–94%)#	85% (81%–88%)
Rutledge & Crouch [19]	185	86% (80%–91%)	-
Baart de la Faille [130]; smear-negative &	597	85% (82%–88%)	75% (71%–78%)
Baart de la Faille [130]; smear-negative%	2484	90% (89%–91%)	-

*As reported by Berg [115];

† In this re-analysis, 1464 of the total of 1787 tuberculosis patients were included, for part of whom 5- and 10-year survival rates could be calculated;

§ Based on 975 cases diagnosed between 1920 and 1930;

‡ These are 534 patients who were smear-positive at the time of discharge from sanatorium and also originally diagnosed as smear-positive;

¶ We only used the period (notified cases between 1928 and 1934) for which the author considered his material to be least biased;

& These 597 patients were once smear-positive but had become smear-negative at the time of discharge from sanatorium;

# 4- instead of 5-year survival;

% These are 2484 patients who were consistently smear-negative but it is unclear how many were culture-positive.

In studies that reported on this (particularly Berg [115] who reports a 30.7% mortality during the first year of follow-up) mortality tended to be highest shortly after diagnosis. This decline in risk with time is also apparent from Table 3 as 10-year survival probabilities tend to be better than the square of the 5-year survival probabilities, as would be obtained with constant mortality rates (risk of dying among those still alive). As cures were not recorded, it is unclear whether this decline is due to a decline in the mortality rate among those still having active tuberculosis, or whether this is due to a decline in the number of people still diseased, so that the denominator gets progressively inflated by cured patients.

Nevertheless, although mortality rates decline, long-term survivorship (of 10 years or more) is much poorer (a 10-year CF of 70% or more) than 5-year survival showing that tuberculosis can be a very long-lasting, chronic disease. Taking the crude unweighted average of all studies one arrives at a 5-year case fatality of 58% and a 10-year case fatality of 73% for open (smear-positive) tuberculosis. Taking an average weighted by sample size these numbers are 55% and 72% respectively. Of course, these mortality data are somewhat distorted by mortality from other causes, as most studies do not record cause of death, and all-cause mortality rates may have been somewhat higher in the pre-antibiotic era than they are now. On the basis of the above data, especially the studies by Berg [115], Thompson [133], and Buhl and Nyboe [131] which – unlike studies on sanatorium patients – appear to be mostly population based, a 30% 10-year survival for smear-positive patients, i.e. a 70% CF, as used by WHO and others in their estimates of the burden of tuberculosis [27], seems a reasonable ballpark figure. As tuberculosis is mostly a disease of young to middle-aged adults the distortion by other causes of death is probably small.

A single, aggregate, CF for all smear positive patients is only justified if in most studies the differences in mortality between the sexes and age groups are rather small. This appears to be the case for sex, but higher ages appear to have somewhat poorer prognosis. For example, in Berg's study (providing the most detailed data), age- and sex specific 10-year mortality rates were 66% for men aged 15–29 years, 70% for men aged between 30 and 49 years, and 94% for men of 50 and older. For women, these rates were 70%, 69%, and 92% respectively [115]. Similar patterns are apparent in other studies providing age (but often using different age-groups) and sex specific mortality.

### Notification and mortality studies

Braeuning [134] reported a ratio of mortality to notification (RMN) for ‘open’ tuberculosis of 70%. This was adjusted for mortality arising from not-previously notified tuberculosis cases by identifying the number of tuberculosis deaths that had been notified as tuberculosis cases previously, but not for changes in either population or incidence over time.

Drolet [116] reported RMNs of approximately 43% for New York City and Detroit, approximately 32% for Chicago, 51%–52% for both New York State and New Jersey, and 55% for Philadelphia. For Massachusetts and England/Wales mortality to notification ratios of 54% were reported. Percentages in all areas were approximately stable over the period for which data are provided, with the possible exception of England and Wales where declines in RMNs were observed. Cases in New York City, Chicago, and England/Wales (from 1923 onwards) also include those first identified from death certificates, all others areas include “primary” notifications only. As this was a period of general decline in tuberculosis incidence, RMNs may slightly overestimate CF as the deaths occur among tuberculosis patients who were incident cases several years earlier and thus the number of deaths in any year would exceed the number of future deaths that would (ultimately) occur among incident cases in that year. In addition, some additional overestimation may be possible if mortality data were more complete than notification data. Pulmonary forms were diagnosed by Z-N smear and chest X-ray and/or clinical symptoms and do not necessarily only include L-J culture-positive cases. The proportion of smear-positive cases was not presented. Variations in CF among regions may well be due to differences in diagnostic methods, reporting systems, inclusion of cases from death certificates, etc., rather than true heterogeneity in prognosis. The only conclusion that stands out from these data is that the prognosis of all forms of (pulmonary) tuberculosis is much better that that of smear-positive cases only.

#### Prevalence and incidence studies

The CF of pulmonary tuberculosis, smear-positive and/or culture-positive, can also be estimated from the NTI study [29] which comprised 4 successive waves of surveys. Diagnosis was by both smear and culture among those with chest radiograph abnormalities. This study reports on: i) prevalence of tuberculosis at each survey, stratified by smear status; ii) the incidence between surveys, i.e. new cases at each survey among those free of tuberculosis at previous surveys, outcome (dead, alive and excreting bacilli, or not excreting bacilli) of prevalent cases at each survey during the subsequent survey; iii) the outcome of prevalent cases at the first survey during all subsequent surveys; iv) the outcome of prevalent cases at each survey during the subsequent survey; v) the outcome of incident cases at each survey at the subsequent survey, i.e. 1.5 years later; vi) the relapse ‘rate’ (which was actually a proportion). The presentation of some of the data in the paper is misleading. Notably, the reported (approximately) 50% 5-year mortality, which is also reported in the abstract of the paper, is incorrect. The reason for this is that loss-to-follow up is inadequately accounted for, and disproportionately affects surviving patients. Once a patient is observed to have died he can no longer become lost to follow-up. A simple comparison of data on the cohort of patients identified at survey 1 (Their Fig. 2. *Fate of cases discovered at the first survey and of patients still excreting bacilli when examined at subsequent surveys*) with data on the fate of patients present at each survey (Their Fig. 3. *Fate of prevalence cases discovered at survey I, II, and III over a period of 1.5 years*) shows this. In Fig. 2 mortality of those discovered at survey I, after 1.5 years is 30.2%, while in Fig. 3 it is 24.7%. Another shortcoming of the paper is that patients without abnormalities on chest radiograph were not examined in this survey and thus not identified. The percentage of pulmonary tuberculosis patients without chest radiograph abnormalities varies between 3 [138] and 50% [139].

There is a better approach to estimating the CF from the NTI data. Ultimately, all tuberculosis patients will either die or get cured. If the ratio of the mortality rate to the cure rate is independent of disease duration, then one can simply look at the ratio of the number of deaths to number of patients cured over a fixed period of follow-up. This assumption seems to be supported by their data (their Fig. 2), as the cured-to-death ratio among the cohort of tuberculosis patients discovered at survey 1 seems to remain about equal at 27.8/30.2, to 23.6/20.0 to 17.2/15.0 in the intervals between survey 1 and 2, between survey 2 and 3, and between survey 3 and 4 respectively. Thus, the prognosis (outcome) of the participants' disease (death or cure) did not seem to depend on the time they had already suffered from tuberculosis. Nevertheless, an exception may have to be made for incident tuberculosis patients who appear to fare somewhat better than prevalent cases, with a cured-to-death ratio of 44/24.

The study reports a total of 428 (often overlapping) individuals alive with tuberculosis at the beginning of any of the 1.5-year intervals. During the subsequent 1.5 years, a total of 89 died and 132 were cured, suggesting an (ultimate) tuberculosis mortality of 40.2%. However, this is not entirely correct as the paper reports 7% mortality among “cured cases” (most presumably dying from tuberculosis) and a “relapse rate” of 10%. We thus subtract 17% from 132 giving 109.5 and add 7% of 132 to 89 giving 98. Thus if the fate of prevalent cases would equal that of incident cases, 47% would ultimately die.

The assumption, as stated above, that there is a constant death-to-cure ratio may not be entirely true, as among follow-up *incident* cases there were almost twice as many cured cases (44) as deaths (24). This ignores relapses. However, as the proportion dying (35%) among those who either die or get cured in these incident cases differs only marginally (and statistically not significantly) from the uncorrected (for relapse) mortality of prevalent cases (40.2% of those who either die or get cured), we seem to be justified assuming a constant death-to-cure rate. Thus our ‘best’ estimate of tuberculosis CF from this study is 47%.

Unfortunately, it is not possible to estimate the CF for smear-positive and smear-negative tuberculosis separately from the data provided. If we accept an ultimate mortality of smear-positive tuberculosis of 70% (based on the studies presented elsewhere in this paper) then assuming that 50% of cases are smear-positive (of all *prevalent* cases, 51% were smear-positive [29], but what counts is *incident* cases which are unidentifiable from their data, so this assumption is questionable) then (ultimate) mortality among smear-negative pulmonary patients would be 24%. Thus the 20% mortality for smear-negative pulmonary tuberculosis, as assumed by WHO and others in their estimates of the burden of tuberculosis (Table 4) seems a reasonable figure.

**Table 4:** Case fatality rates used by the WHO to provide estimates of burden of disease*.

Category	CFR (%)	Region to which CFR is applied
**HIV negative**		
smear-positive untreated	70%	Global
smear-negative untreated	20%	Global
**HIV positive**		
smear-positive untreated	83%	Global
smear-negative untreated	74%	Global

*WHO: World Health Organization; CFR: case fatality rate.

### Analysis of Duration of Disease

The duration of disease is the time from onset of disease till cure or death. For tuberculosis, it is not possible to measure exactly when it started, as patients may remain asymptomatic or have very mild symptoms shortly after getting the disease. Moreover, of the two possible end points, cure is hard to measure, as relapses are common [140] and establishing cure in untreated tuberculosis patients requires extensive medical investigations. No single study reports on the duration of disease by systematic follow-up of incident cases so we had to estimate duration indirectly.

#### Prevalence and mortality studies

Duration of disease can be estimated indirectly from the ratio of prevalence to mortality. The Framingham Community Health and Tuberculosis Demonstration [20], [135]–[137] reported a presence of approximately 9 active (presumably a combination of smear-positive, smear-negative culture-positive, and other forms tuberculosis) living cases to every death, and 3 smear-positive cases for every death. Assuming a long term mortality of 70% among smear-positive and 16% mortality among all others (i.e. assuming that active smear-negative cases are similar to Krebs' closed tuberculosis [121], as both presumably included cases with only chest radiograph abnormalities in addition to culture-positives) one obtains a CF of 0.34, and an average duration of 3 years. On the basis of this study it is impossible to stratify by smear and culture status.

#### Prevalence and incidence studies

The duration of disease in the pre-chemotherapy era was only studied prospectively in one other study, *viz.* the NTI study [29]. As follow-up of prevalent cases does not provide reliable data about duration of disease, the best approach to estimate this parameter would be the prevalence-to-incidence ratio which is (almost) 4. This is very close to the ratio found for bacillary (i.e., sputum and/or culture positive) pulmonary tuberculosis in New Delhi, India over the period 1962–1970 [113] using similar methodology as the NTI study. Unfortunately, availability of treatment, affecting the duration of disease, was not reported on; therefore, we cannot include the study to estimate the duration of untreated tuberculosis.

As waves of surveys in the NTI study were 1.5 years apart (even 2 years for the interval between wave 3 and 4) [29], one has to adjust for missed incident cases, i.e. for the incident cases who recovered, migrated out or died before being detected in one of the surveys. If we would assume an exponential duration of disease with parameter δ (the inverse of the duration of disease), then in an interval of length T (1.5 years) we would observe a fraction (1-exp(-δT))/(δT) of the intervening incident cases at the following survey. Under these assumptions an average duration of 3.33 years (i.e. δ = 0.3) would fit the NTI data almost perfectly. Perhaps, the number missed between surveys may be slightly larger due to non-exponential survival (specifically, incident cases recovering or dying on average faster than prevalent cases). If so, 3.3 years would slightly overestimate the duration of disease. We infer that an average duration of approximately 3 years of smear-positive and smear-negative cases combined would seem the most plausible estimate.

There is almost no reliable information regarding the relative duration of smear-positive and smear-negative tuberculosis disease. A study from South India [141] provides some insight in the natural duration of smear-positive tuberculosis as the authors give the ratio between incidence and prevalence for these patients. They estimated a ratio of 0.46 corresponding to an average duration of 2.2 years. This is considerably shorter than the mean duration estimated in the NTI study in Bangalore for the mix of smear-positive and smear-negative patients, suggesting a much shorter duration for smear-positive than for smear-negative patients. However, as the study was carried out in the 1980s it seems likely that the average duration must have been shortened by available chemotherapy (INH plus thiacetazone), as was also suggested by the authors of the paper. This is also supported by another study carried out in South India [142] where the incidence of culture-positive tuberculosis was 1,578 and that of smear-positive culture-positive tuberculosis 726/100,000 (V. Kumaraswami, personal communication), supporting the assumption that approximately 50% of both incident *and* prevalent cases of culture confirmed tuberculosis are smear-positive. Overall this seems to support the notion that the natural duration of smear-positive and smear-negative disease are roughly similar.

## Discussion

### Main findings

In our study we combined available information on untreated tuberculosis to estimate its case fatality and duration of disease. We found only few studies from the pre-chemotherapy era that allow for estimation of CFs and duration of disease of smear-positive tuberculosis. Given the limited information available and assuming that a 10-year CF will closely approximate lifetime CF, we conclude that (lifetime) CF in untreated smear-positive tuberculosis among HIV negative individuals is approximately 70% and about the same for both sexes. Mortality seems to be approximately independent of age until the age of 50 years after which it increases, perhaps due to concomitant complicating diseases such as diabetes or cancer and a greater mortality from other causes. However, this age effect would only be important in (patient) populations with a dramatically different age structure than the ones used in this review. For most high burden countries this is not the case.

For culture-positive smear-negative tuberculosis, lifetime CF is probably slightly over 20%, although this could only be estimated indirectly and with uncertain precision.

The duration of tuberculosis from onset to cure or death is approximately 3 years and appears to be grossly similar for smear-positive and smear-negative tuberculosis.

Because of the expected heterogeneity between studies with respect to study design and population, study period, duration and intensity of follow-up, definition of pulmonary tuberculosis (‘open’/‘closed’, bacillary/abacillary, smear-positive/smear-negative), etc., we did not do a formal meta-analysis. Additional heterogeneity among studies may also exist in patient selection and diagnostic procedures, for example the number of sputum samples analyzed and how these were obtained (e.g. induced or spontaneous). However, these data were hardly ever reported in the included studies.

### Limitations of our systematic review

Despite the fact that (HIV negative) tuberculosis has for centuries been a major cause of mortality, the number of studies on its natural history is surprisingly low.

This contrasts sharply with, for example, HIV for which detailed information on its natural history became available within decades of the discovery of the virus. Long term follow-up studies of HIV patients in carefully monitored cohorts have generated this information. In contrast, follow-up of most tuberculosis patients is nowadays usually limited to the duration of their treatment.

Another limitation is our serious lack of knowledge on the prognosis of extra-pulmonary and smear-negative pulmonary tuberculosis as most data on the natural history are available for patients who tested sputum smear-positive. No reliable prospective data on smear-negative culture-positive pulmonary patients are available and their long term survival can only be estimated indirectly and thus with great uncertainty. These patients form currently the group most likely to receive no or inadequate treatment, and may well account for large proportion of tuberculosis deaths. The prognosis of untreated extra-pulmonary patients - a very heterogeneous group that also includes most tuberculosis in children - is even more uncertain, and insufficient data were identified to include it in our review.

An important limitation of using electronic databases going back in time is that these do not include abstracts and searches therefore may miss potentially eligible papers. We have tried to obviate this by including quite general search terms (see Table 1). However, this way of searching yielded many references (n = 1560), 43 of which were selected for reading and available in full-text, but none of which was eligible for inclusion into our review.

We therefore supplemented our search strategy with snowball sampling. A limitation of this approach is that it depends, perhaps heavily so, on its starting point. We choose dr. Rieder's book [18] as the starting point since it is known for its thoroughness with respect to discussing all important aspects of tuberculosis and inclusion of (older) literature. Although this approach may have lead to some underrepresentation of e.g. American and francophone literature, this latter strategy yielded 22 eligible papers whereas the electronic searches did not yield any useful references.

Quite some of the identified potentially eligible papers were not available to us. In theory, this may have influenced the outcome of our review. However, we were able to identify papers appearing in a variety of journals, text books and published as reports (‘grey literature’) and did not find any evidence for a correlation between the type of source and the quality of the data. Therefore, we expect no important ‘availability bias’ correlated with prognosis of untreated tuberculosis.

Another limitation of our review is that most of the included studies on CF were on predominantly Caucasian populations whereas most untreated patients currently are of different ethnicity. This is probably mainly due to the fact that evaluating the natural history of tuberculosis requires long term follow-up which has proven to be difficult, especially in resource constrained settings.

A key limitation is that we had to restrict our review to HIV-negative patients, as explained in the introduction. This does not imply that no information on the prognosis of tuberculosis in HIV-positive patients is available. For example, two relevant systematic reviews have been carried out recently: one on any form of tuberculosis in people with HIV infection [143], and one on HIV and MDR-tuberculosis [144]. The prognosis of the latter type of patients likely resembles that of untreated patients. If we exclude data on patients receiving ART, because of the heterogeneity in ART regimes and ART resistance patterns - both between and within countries, then we can at least explore the prognosis of HIV co-infected tuberculosis patients. As regards CF, the review of Payne and Bellamy [143] provided no information on the prognosis of HIV positive MDR-tuberculosis patients. However, it identified several sources on tuberculosis in HIV patients from the pre-ART era. One from the USA found a median survival of tuberculosis patients, including patients with drug susceptible tuberculosis, of 16 months [145]. However, only 13% of patients died from tuberculosis, the others from other AIDS related diseases. Development of tuberculosis may thus be a marker for being severely immunocompromised. Another study, from Malawi, found a mortality of 47% among patients followed-up for 32 months [146]. Thus, HIV infected patients with tuberculosis not treated with ART, have a poor prognosis. The other review [144] identified 8 sources of HIV associated MDR-tuberculosis outbreaks. Five of these were from the USA where second line tuberculosis treatment is presumably available and adequate, and these studies thus did not represent “untreated” tuberculosis. This also appeared from case fatality rates which were lower than those from outside the USA. The other three studies were from Italy (N = 116) [147], Spain (N = 48) [148], and Argentina (N = 124) [149]. The studies from Italy and Argentina both reported that second line treatment was not adequate, while the use of second line drugs was not reported in the study from Spain. Reported mortality was between 93% (Argentina) and 98% (Spain), and time to death was short. In the Spanish study the mean time from diagnosis to death of the 47 who died was 77.6 days [148]. In the Italian study the median time to death was reported as 93 and 79 days for the two participating hospitals [147]. The Argentinean study only reported that half of the patients survived less than 5 weeks and that 1-year survival was as low as 5% [149].

As regards the duration of disease, findings from these studies [143], [144], [146]–[149] suggest that untreated tuberculosis in HIV infected patients must be rapidly fatal, with a mean survival of less than 6 months. However, a limitation of the use of these patients is that all suitable reports were on nosocomial outbreaks among hospitalized HIV patients. Such patients may be more immunocompromised than the “average” HIV patient who develops tuberculosis, and alternative approaches to estimate the prognosis of tuberculosis in various types of HIV infected patients should be developed.

### Conclusions

While pre-chemotherapy data appeared to be a useful source of data for the prognosis of untreated tuberculosis, inevitably questions remain. Particularly, the impact of risk factors other than (variably defined) smear status was hard to explore systematically. Perhaps, long-term follow-up of patients with inadequately treated MDR or XDR tuberculosis may fill some of the gaps in our knowledge. Such follow-up may also fill other gaps in our knowledge such as the frequency of transitions between smear-positive and smear-negative tuberculosis and the prognosis and duration of HIV-positive tuberculosis.
